# Human Polymorphonuclear Cells Support Zika Virus to Cross Endothelial Monolayer and Access Bloodstream

**DOI:** 10.3390/pathogens11030321

**Published:** 2022-03-05

**Authors:** Claudia Gandolfo, Chiara Terrosi, Shibily Prathyumnan, Gabriele Anichini, Gianni Gori Savellini, Giuseppe Morgante, Maria Grazia Cusi

**Affiliations:** 1 Department of Medical Biotechnologies, University of Siena, 53100 Siena, Italy; claudia.gandolfo@unisi.it (C.G.); chiara.terrosi@unisi.it (C.T.); shibily.prathyumn@student.unisi.it (S.P.); gabriele.anichini@student.unisi.it (G.A.); gianni.gori@unisi.it (G.G.S.); 2 Department of Molecular and Developmental Medicine, University of Siena, 53100 Siena, Italy; giuseppe.morgante@unisi.it

**Keywords:** Zika virus, HUVECs, viral infection, cell junctions, endothelial monolayer

## Abstract

The rapid spread of new outbreaks of human infection caused by Zika virus (ZIKV) has raised many global concerns since 2016. Despite the increasing knowledge of this virus, data on the pathogenesis of ZIKV are still missing. In particular, it is still unknown how the virus crosses the endothelial monolayer and gets access to the bloodstream. In the present work, we used human umbilical vein endothelial cells (HUVECs) as a model to study ZIKV infection *in vitro*. We demonstrated that HUVECs are an optimal reservoir for viral replication, as they were able to sustain ZIKV infection up to two weeks, without showing a cytopathic effect. In order to evaluate the integrity of endothelial monolayer, immunofluorescence was performed on mock-infected or ZIKV-infected cells ± peripheral blood mononuclear cells (PBMCs) or polymorphonuclear cells (PMN), 48 h p.i., by using an anti-VE-Cadherin antibody, a major adherence protein that maintains the integrity of intercellular junctions. In addition to infection, we noted that the presence of some components of the immune system, such as PMNs, played an important role in altering the endothelial monolayer in cell junctions, suggesting that presence at the site of infection probably promotes the spread of ZIKV in vivo in the bloodstream.

## 1. Introduction

Zika virus (ZIKV) is a re-emerging arthropod-borne virus (arbovirus) belonging to the *Flaviviridae* family [1,2]. ZIKV was first isolated in a sentinel Rhesus monkey in Uganda in 1947. Outside this zone, it spread eastward to French Polynesia in 2013–2014, reaching Latin America in 2015 and disseminating in North America in 2016. Currently, imported cases of Zika fever have been reported in travelers returning from areas with endemic/epidemic Zika fever, increasing the risk of virus dissemination where its vectors, such as *Ae. aegypti* and *Ae. albopictus*, are present. This RNA virus is closely related to other members of the genus, including Dengue Virus (DENV), West Nile virus (WNV), Yellow Fever Virus (YFV), Tick-Borne Encephalitis Virus (TBEV), and Japanese Encephalitis Virus (JEV) [3]. The positive sense single-stranded RNA genome of ZIKV consists of a single polypeptide encoding an open reading frame, flanked by noncoding regions. Viral and host proteases subsequently cleave the viral polypeptide (5′-C-prM-E-NS1-NS2A-NS2BNS3-NS4A-NS4B-NS5-3′) into seven non-structural proteins (NS1, NS2A, NS2B, NS3, NS4A, NS4B, NS5) along with three structural proteins, the envelope (E), premembrane (prM), and capsid (C). E is the receptor-binding protein, as well as the fusion protein, while prM mainly functions as a chaperone for the E protein to avoid premature fusion [4,5]. The dimeric basic capsid (C) protein combines Zika nucleic acid to nucleocapsid structure. Regulation of viral replication and transcription along with the stimulation of host antiviral responses are regulated by non-structural proteins [6,7]. Based on the phylogenetic analysis of the entire ZIKV genome, previous studies have reported the existence of two genetic lineages of ZIKV that correspond to Asian and African geographical regions [8]. Data on the pathogenesis of ZIKV are scarce, as it has received far less attention than other arboviruses, such as YFV, DENV, WNV, JEV, and CHIKV. Like many other members of the *Flaviviridae* family, ZIKV is transmitted by the bite of *Aedes* mosquitoes [9,10]. However, the mechanisms of ZIKV infection, the signaling pathways and antiviral immune response of the host elicited by this virus, remain to be determined. Due to the capacity of mosquitoes to inoculate ZIKV into the human skin during the blood-feeding process, potential target cells for infection with this virus are likely to be localized on the epidermis and dermis, which also constitute the first line of defense. Skin fibroblasts and epidermal keratinocytes are found to be highly permissive for ZIKV infection [11]. Different cell types, such as epidermal keratinocytes [12], dendritic cells [13] or neurons [14], are targets of flaviviruses. Compared to other flaviviruses, ZIKV is characterized by its unique ability to be sexually transmitted, to cross placental and blood-brain barriers and to cause microencephaly in utero [15]. Cellular tropism studies have reported that neural stem cells, astrocytes, oligodendrocyte precursor cells, and microglia are preferentially infected by ZIKV. Reports indicate that ZIKV actively infects placenta and its barrier cells, namely trophoblasts and fetal endothelial cells [16,17,18]. A mechanism that viruses use to cross the placental barrier is the permeabilization of the endothelium [19,20]. Endothelial cells (ECs) are also major components of the blood-brain barrier and part of the placental blood barrier, by preventing the circulating virus from entering both the brain and the fetal tissues [21]. Recent studies have demonstrated that fetal endothelial cells or human umbilical vein endothelial cells (HUVEC) are permissive for ZIKV infection, making HUVEC a key cell model for ZIKV studies [22]. At present, it is unclear how ZIKV reaches immune-privileged sites within the body and breaches protective placental-fetal and blood-testis barriers, which leads to sexual transmission and congenital defects [23]. A possible route is represented by ZIKV-infected white blood cells (WBCs), which disseminate the virus into different compartments of the body or serve as a viral reservoir after recruitment to the infection site similarly to DENV. Studies have shown that WBCs are permissive for ZIKV infection, whose main target are monocytes, particularly CD14 + CD16 + monocytes followed by DCs. As monocytes can infiltrate many tissues, including immune-sheltered organs, they are ideal targets for infection [24].

In the present study we used HUVECs as a model to understand the mechanism(s) utilized by ZIKV to cross the endothelial monolayer and get access to bloodstream. We investigated whether the infection alone altered the endothelial monolayer in cellular junctions, or whether the presence of WBCs increased the permeability of the monolayer, by favoring virus release.

## 2. Results

### 2.1. Survival of the Virus

**Figure 1:**
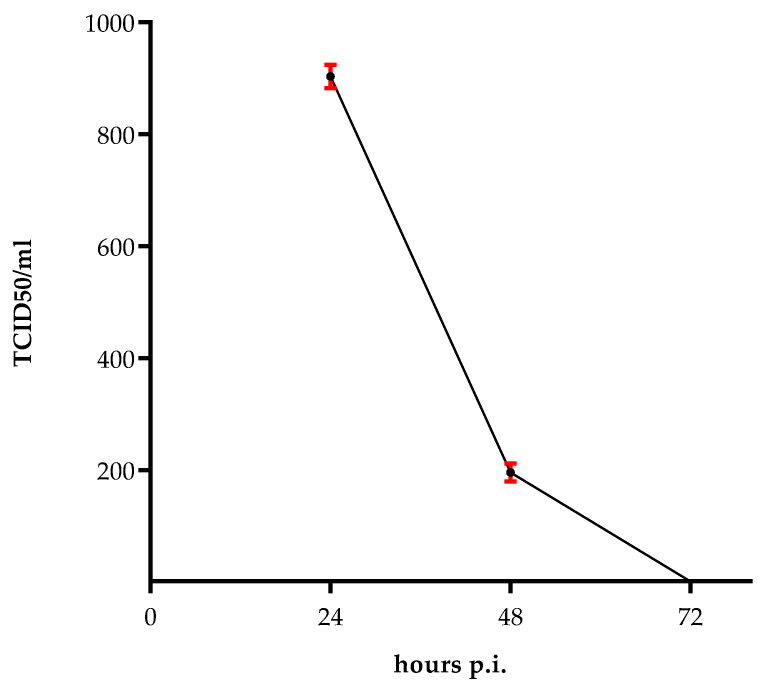
Survival of ZIKV in the environment. Viral titration after incubation of ZIKV at 37 °C for 24, 48 and 72 h. Data are representative of three independent experiments and are expressed as mean ± SD.

To determine the survival of the virus in the absence of cells, at a mean temperature of the areas where the virus vector is circulating, we left 100 µL of virus (6.3 × 10^7^/mL TCID_50_) at 37 °C for 24, 48 and 72 h. Afterwards, the virus was titrated on Vero cells. There was a significant reduction in viral over time with 9.2 × 10^2^/mL TCID_50_ at 24 h post infection, and 2.2 × 10^2^/mL TCID_50_ at 48 h post infection, and no live virus survived after 72 h (Figure 1).

### 2.2. Persisting ZIKV Production in HUVECs

**Figure 2:**
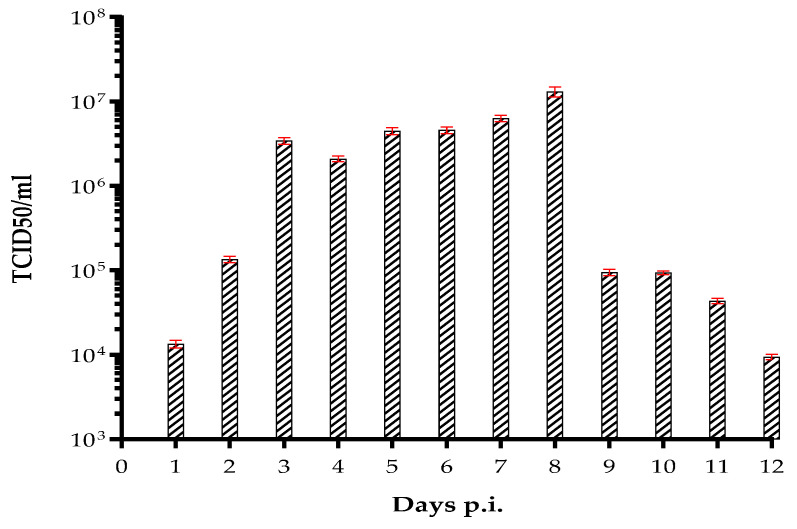
Evaluation of ZIKV replication in HUVECs for 12 days. Viral titration of the supernatant collected from HUVECs, infected with 0.1 MOI. The mean values of at least three sets of experiments ± SD are presented.

The viability of the virus used to infect HUVECs was confirmed by titration of the supernatant collected from the infected cells for 12 days. An increase in viral titer was observed in supernatants collected up to 8 days p.i. (1.2 × 10^7^/mL TCID_50_), when the virus reached a peak, followed by a gradual decrease up to day 12 (9.2 × 10^3^/mL TCID_50_) (Figure 2). Despite the virus was still present at day 12, it was not possible to evaluate it for a longer time, as endothelial cells are short-lived cells. At that time, the viability of the mock-infected cells was evaluated by trypan blue staining, as described in Mat&Met.

### 2.3. HUVEC Permeabilization after ZIKV Infection

**Figure 3:**
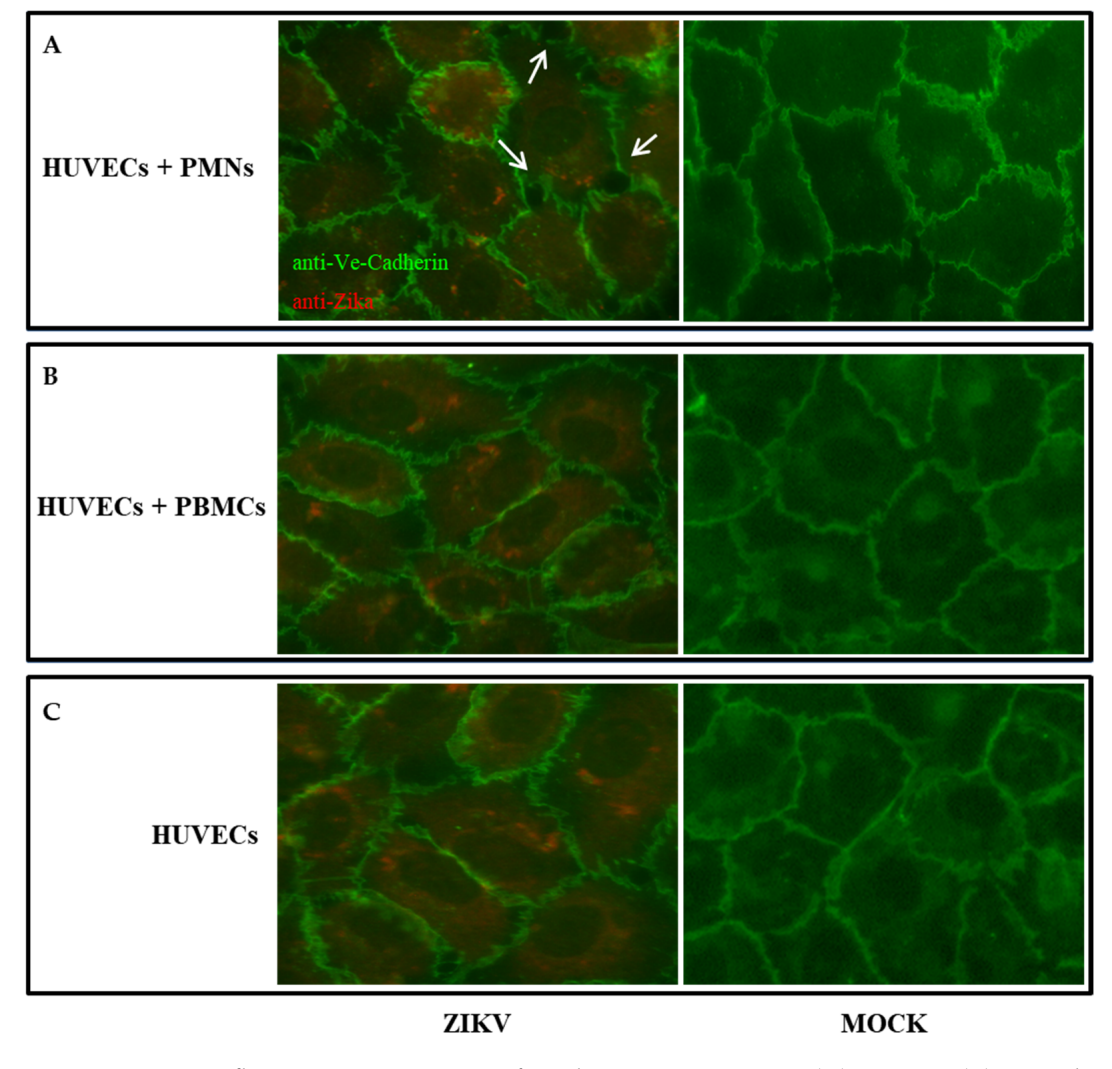
Immunofluorescence on ZIKV-infected HUVECs + PMNs (**A**), PBMCs (**B**) or without WBCs (**C**), 48 h p.i. and the corresponding mock-treated samples. The cells were stained with anti-Flavivirus (4G2 antibody), followed by anti-mouse IgG-AlexaFluor 594; and with anti-VE-cadherin, followed by anti-rabbit IgG-FITC. Arrows indicate endothelial cell borders with damaged VE-Cadherin. Magnification 200×.

We analyzed whether ZIKV might permeabilize the monolayer of HUVECs in the presence of PBMCs or PMNs. HUVEC monolayer on a transwell plate was infected with 0.1 or 0.5 MOI of ZIKV for 90 min. Five hours post infection, the medium was removed and freshly isolated PBMCs or PMNs were added to the monolayer. The integrity of the monolayer was determined 48 h p.i., by adding FITC-Dextran to the upper layer of the transwell. Although the addition of PMNs appeared to modify the integrity of the monolayer compared to the control, as shown in Figure 3, no significant permeability of the monolayer was observed in all samples, in comparison with the mock infected cells. In addition, a virus titration was carried out in the medium collected from the upper and lower compartments of the infected endothelial cells before the addition of FITC-Dextran. The virus was detected in the upper compartments of all infected cells, alone or in the presence of PBMCs or PMNs. The highest titer was obtained in infected HUVECs + PMNs with 0.1 MOI (2 × 10^3^/mL TCID_50_). In the lower compartments, the virus was only revealed in HUVECs + PMNs with 0.1 MOI, at a very low amount (6.3 × 10^2^/mL TCID_50_; *p* = 0.006).

### 2.4. The Presence of PMNs Influences the Permeability of the Endothelial Monolayer

ZIKV can infect many cell types and causes cytopathic effect (CPE) on Vero cells as well as neurons; however, it does not elicit cytopathology in brain microvascular endothelial cells [25]. In this study, we confirmed that HUVECs are able to sustain the replication of ZIKV; however, they do not present morphological changes upon infection, as previously described [25]. Indeed, as observed 48 h p.i., the infected cells did not show any cytopathic effect, as confirmed by the cytotoxicity test, which showed a 72% viability (LDH release 28% ± 0.75) compared to the 80% viability (LDH release 20% ± 0.51) of the control (*p* > 0.05). Moreover, in order to evaluate the integrity of endothelial monolayer, immunofluorescence was performed on mock-infected or ZIKV-infected cells ± PBMCs or PMNs 48 h p.i., by using an anti-VE-Cadherin antibody, a major adherence protein that maintains the integrity of intercellular junctions (Figure 3). Our analysis revealed that during infection, the presence of PMNs influenced the integrity of the endothelial monolayer, resulting in an alteration of the cell-cell adhesion and increased permeability, as shown in Table 1 (*p* = 0.03).

### 2.5. Cytokine Profile

Among the cytokines tested in the supernatant of the upper compartment of transwells, containing mock-infected or ZIKV-infected HUVECs ± PBMCs or PMNs, IL-1β, IL-6 and TNF-α and GM-CSF were only detected in ZIKV-infected cells + PMNs. Among the pro-inflammatory cytokines tested, IL-6 significantly increased to 162.92 pg/mL, compared to 47.99 pg/mL in the corresponding control (*p* = 0.02). A very modest amount of IL-1β and TNF-α was detected, as shown in Table 2. GM-CSF (4.51 pg/mL) was only detected in ZIKV-infected HUVECs + PMNs. None of the other samples showed any change in the concentration of the tested cytokines.

## 3. Discussion

The re-emergence of Zika virus has been raising global concerns since 2016, due to its rapid spread worldwide and clinical manifestations in humans. ZIKV can be found in a variety of body fluids, such as tears, saliva, semen, cervical mucus, and urine during human infections [26,27,28,29,30]. Despite the infection is usually caused by close contact with infectious body fluids, a case of ZIKV infection had been reported in 2016 without any documented mosquito bites or contact with infectious body fluids [31]. The survival of ZIKV in the environment is a well-known phenomenon in the epidemiology of viral pathogens. Since structural stability of viruses may aid their survival in harsh conditions, we studied the persistence of ZIKV in a cell-free environment. Although the virus was reported to be structurally stable at 40 °C for up to 60 min [32], we observed a decrease in ZIKV infectivity in Vero cells when pre-incubated at 37 °C for 72 h (Figure 1). Furthermore, we found that ZIKV was able to survive after 48 h. This indicates that a proper surface disinfection would be necessary in order to limit its transmission. However, once in the body, ZIKV can persist in body fluids for months [33] and is able to persistently infect endothelial cells for nine days [15]. In our study, we confirmed these data and demonstrated that the virus is able to persistently infect endothelial cells for two weeks. Since HUVECs are short-lived cells, it was not possible to analyze virus production from cells older than 12 days. Nevertheless, we established that the virus titer was >10^3^/mL at the last culturing day, indicating that the virus is capable of replicating in these cells for a longer time. This suggests that ZIKV-infected HUVECs could act as a reservoir for viral replication and sustain the viral spread. Indeed, it was reported that, subsequent to infection of skin fibroblasts and epidermal keratinocytes in the epidermis and dermis [11], the virus, transmitted by a mosquito bite, is able to infect endothelial cells and release viral particles via hematogenous dissemination. Therefore, in order to understand how ZIKV can enter the bloodstream, we analyzed the in vitro infection of HUVECs, in the presence of different populations of white blood cells. The virus was released from the apical surface of polarized HUVECs, but failed to alter their permeability, as it was already observed by others [15,25]. Permeabilization of the endothelium is a mechanism by which flaviviruses can bypass the endothelial cell barriers [34]. We found no significant difference in the permeability of the HUVEC monolayer among the ZIKV and mock-infected cells in a transwell system. However, we did notice that the addition of PMNs to ZIKV-infected HUVECs seemed to influence the endothelium consistency, compared to the control. We found a modest virus titer (2 × 10^3^/mL TCID_50_) in the upper compartment of the transwell, and a very low amount (6.3 × 10^2^/mL TCID_50_; *p*=0.006) in the lower compartment. This was contrary to what observed by Alimonti et al. [35], who reported the basolateral release of the virus from brain microvascular endothelial cells (BMECs). An explanation for this could be the morphological and functional differences that exist among BMECs and HUVECs [36,37]. Moreover, we observed neither a cytopathic effect nor a significative disruption of tight junctions on infected HUVECs two days p.i. These results are in line with previous studies performed on human brain microvascular endothelial cells (HBMECs), in which ZIKV reached CNS without disrupting the endothelial barrier [15,25].

A regulated, autophagy-like mechanism has been reported in ZIKV-infected cells. This could explain the possible existence of other mechanisms by which the viral particles are released from the infected cell [11,38,39], apart from cell permeabilization or disruption. A study reported that, during viral replication, new ZIKV particles were individually and evenly released in small exocytic vesicles over a large surface of the cells, instead of clusters. This suggests that secretory autophagy could be involved in virion release, by leaving the cell apparently undamaged [40,41].

Furthermore, our aim was to understand what occurs when WBC populations are recruited by ZIKV-infected endothelial cells. To this purpose, we also investigated whether the addition of PBMCs or PMNs could alter the production of cytokines on ZIKV-infected HUVECs, since these play a crucial role in the regulation of vascular permeability and the control of hemostasis [42]. Our data show that, compared to mock-infected cells, the addition of PMNs to ZIKV-infected HUVECs resulted in a significant increase in pro-inflammatory cytokine level, such as IL-6 and, to a lesser extent, IL-1β and TNF-α (Table 2). In particular, IL-1β and TNF-α, produced by infiltrating inflammatory cells, can induce endothelial cells to express several cytokines, such as IL-6, and contribute to the endothelial leaking. Therefore, when HUVECs are infected with ZIKV, leukocytes, recruited by the endothelium at the site of injury, tether to and roll on its surface, leading to endothelium damage. This phenomenon could explain the presence of the virus in the lower compartment of transwell chamber, only when PMNs were added to infected HUVECs. A very low amount of GM-CSF was also expressed in response to TNF-α, promoting inflammatory cell adhesion to the endothelium and potentially assisting in inflammatory cell transmigration. These features were confirmed by immunofluorescence, which showed a partial alteration of VE-cadherin in the cell structure of infected HUVECs + PMNs only. These results suggest that most of the viral particles are released as small exocytic vesicles in the apical side of the endothelial layer. However, a lower amount of virus is released as a consequence of a modification of endothelial VE-cadherin structure in the presence of PMNs mediating cell permeability.

## 4. Materials and Methods

### 4.1. Cells and Virus

Vero (ATCC CCL-81) cells were grown as a monolayer in Dulbecco’s modified Eagle’s medium (DMEM) (Lonza, Milan, Italy), supplemented with 5% heat-inactivated fetal calf serum (FCS) (Life technologies, Milan, Italy) and 100 U/mL penicillin/streptomycin (Euroclone, Milan, Italy) at 37 °C. Human umbilical cord was obtained from healthy women who underwent uncomplicated term pregnancy (authorization HUVEC2016_16/03/16 approved by Ethical Committee of Tuscany Region, South-Eastern Area). After collection, the umbilical cord was rapidly immersed in sterile saline solution (0.9% NaCl) and immediately processed for endothelial cell isolation and cultured, as described [43]. Briefly, umbilical veins were cannulated at both ends and washed through with PBS. The vein was incubated with 200 U/100 mL of collagenase from *Clostridium histolyticum* (Sigma, Milan, Italy), prepared in Hank’s buffer (Euroclone Italy), at 37 °C for 15 min. Afterwards, cells were washed through the veins, then centrifugation was used to pellet them. Cells were then grown in endothelial growth medium (EBM-2) (PromoCell, Heidelberg, Germany) containing 100 U/mL penicillin/streptomycin, supplemented with 20% FBS and endothelial growth factor supplements (PromoCell, Germany). Cells were maintained at 37 °C with 5% CO_2_. HUVECs cultured for two or three passages were used for the experiments. To assess HUVECs viability, cells were stained with trypan blue (GIBCO) and then counted to determine the percentage of dead cells, as described [44].

Human peripheral blood mononuclear cells (PBMCs) and human polymorphonuclear leukocytes (PMNs) were isolated from a buffy coat of a healthy donor upon informed consent. This research was carried out according to the principles of Helsinki declaration. Ethical approval was obtained from the local ethical committee for clinical trials (authorization TOSV2016_19/04/2016 from Comitato Etico Regione Toscana-Area Vasta Sud Est) in terms of General Data Protection and Regulation (GDPR) upon written informed consent, signed by all subjects prior to participating in this study [45,46]. PBMCs were obtained by Ficoll-Hypaque gradient separation with Lymphoprep (Fresenius Kabi Norge, AS) and resuspended in RPMI-1640 medium (Euroclone, Italy) supplemented with 10% FCS. PMNs were separated by Ficoll 400 and Dextran T 500 methods [47]. PMBCs and PMNs were then analyzed by cytofluorimeter BD LSRFortessa X20 flow cytometer (BD Biosciences), using FICT-conjugated anti-CD3 mAb (eBioscience), FICT-conjugated anti-CD19 mAb (eBioscience), FICT-conjugated anti-CD14 (eBioscience), FICT-conjugated anti-CD11c and APC-conjugated anti-CD66 mAb (eBioscience). Data analysis was performed by using FlowJo v10 (TreeStar, Ashland, OR, USA). Both cell populations were respectively pure at ≥90% and 80%.

Asian strain of ZIKV (Accession Number KJ77679) stocks were prepared by inoculating the strain into confluent Vero cells. Viral infection was confirmed by the presence of cytopathic effect (CPE). Culture supernatant containing the virus was collected and stored at −80 °C.

### 4.2. ZIKV Replication in HUVECs along Time

Twenty-four wells containing HUVECs were prepared and infected with 0.1 MOI of ZIKV at 37 °C for 1 h. Then, cells were washed with PBS and cultured in the EBM-2 medium with 5% of FBS for 12 days. Each day, supernatant was collected from two wells and tested for the presence of viral particles by titration; this procedure was carried out for twelve days. This experiment was repeated three times.

### 4.3. Virus Titration

Virus stocks were titrated by 10-fold serial dilution and inoculated onto Vero cell monolayers. The viral titers were determined by 50% tissue culture infective dose (TCID_50_) assays, using the Reed and-Muench method [47]. After a 5-day incubation, CPE was observed by microscopic analysis and virus titration.

### 4.4. FITC Dextran Permeability Assay

Permeability assays were performed on 24-well plates with polycarbonate filters (3 μm pore and 6 mm diameter (Corning Life Sciences, The Netherlands), coated with HUVECs (5 × 10^4^ cells per cm^2^) in 300 μL of complete EBM-2 medium (upper compartment) and cultured for two days until confluent. Lower compartments contained 700 μL of medium. Before the permeability assay, HUVECs were washed with medium, then infected with ZIKV in triplicate at an MOI of 0.1 and 0.5 for 90 min and incubated at 37 °C for 5 h. Afterwards, the medium was removed and human PBMCs (2 .5 × 10^4^) or PMNs (2 .5 × 10^4^), previously isolated as described above, were added to each well, and incubated at 37 °C for further 48 h. Then, FITC-dextran (4 kDa mol wt, Sigma, Milan, Italy) (0.5 mg/mL) was added to the upper chamber of the filter and, after 3 h, the fluorescence intensity of transmigrated FITC-dextran was measured in the upper and lower chambers by using a Perkin-Elmer fluorimeter (490-nm excitation, 530-nm emission). Uninfected HUVECs, uninfected HUVECs  +  PBMCs, and uninfected HUVECs + PMNs were used as controls. The assay was repeated three times. The FITC dextran transmigrated across the membrane was calculated as the ratio of fluorescence units/mL in the upper to the lower chambers for all experimental samples. In order to monitor ZIKV infections, endothelial cell monolayers in 96-well plates were infected at the same MOI as the transwell plates. Cells were fixed and tested for the presence of virus by immunofluorescence 48 h post-infection.

### 4.5. Cell Viability Assay

Cytotoxicity was assessed by using the CytoTox 96^®^ Non-Radioactive Cytotoxicity Assay (Promega Corporation, Madison, WI, USA) in order to determine the number of viable cells after the infection, according to the manufacturer’s protocol. Absorbance was read at 490 nm. Percentage of cell proliferation was determined by using the following formula:

Percent cytotoxicity = (Experimental LDH Release/Maximum LDH Release) × 100.

### 4.6. Analysis of Endothelial Cell Adhesion Protein by Immunofluorescence

HUVECs were cultured onto a chambered coverslip with four wells and mock-treated or infected with 0.1 MOI, as previously described. After 5 h at 37 °C, human PBMCs or PMNs were added. Forty-eight hours p.i., cells were fixed with 4% paraformaldehyde (J.T: Baker, Milan, Italy) in PBS for 10 min, washed with PBS/0.1% Triton X-100 (Sigma, Milan, Italy) for 10 min. Cells were incubated for 60 min with rabbit anti-human CD144 antibody against VE-Cadherin (E-Bioscience) and with mouse anti-Flavivirus group antigen antibody, clone D1-4G2-4-15 (Absolute Antibody, Italy) as primary antibodies. Cells were washed with PBS, before adding FITC-labeled anti-rabbit IgG (Sigma, Milan, Italy) and AlexaFluor 594-labeled anti-mouse antibody(Abcam, Italy) for 45 min. Cells were then washed in PBS three times and observed under a Leitz Diaplan fluorescence microscope.

### 4.7. Cytokine Analysis

Supernatants from the upper compartments of samples infected with 0.1 MOI in transwell experiments were collected at 48 h p.i. and frozen at −80 °C. Eleven different cytokines, namely GM-CSF, IFN-gamma, IL-1β, IL-12p70, IL-13, IL-18, IL-2, IL-4, IL-5, IL-6 and TNF-α, were analyzed from the supernatants. Cytokine profile was measured by using a ProcartaPlex multiplex immunoassay (Invitrogen, by Thermo Fisher Scientific, Milan, Italy), a magnetic bead-based assay, according to the manufacturer’s instructions. Afterwards, it was read through a Bioplex Magpix Multiplex reader (Bio-Rad Laboratories, Hercules, CA, USA), using Luminex-200 software (Luminex Corporation, Austin, TX, USA). Briefly, supernatants were mixed with beads coated with antibodies (Abs) to various cytokines having unique fluorescent intensity. Subsequently, mixtures were incubated with biotinylated anti-cytokine Abs. Finally, PE-conjugated streptavidin was added, and fluorescent signals were detected by using the multiplex array reader. Raw data were initially measured as a relative fluorescence intensity and then converted to cytokine concentration, based on the standard curve generated from the reference concentrations supplied in the kit. Concentration of cytokines was expressed as pg/mL.

### 4.8. Statistical Analysis

All experiments were carried out in triplicate. Statistical significances were assessed with a two-tailed chi-squared test. Results were considered statistically significant at *p* < 0.05. All analyses were performed by using Graph Pad Prism software (v.7.0).

## Figures and Tables

**Table 1:** FITC Dextran Permeability Assay.

Permeability Assay	
Samples	upper/lower
HUVECs	7.147 ± 0.53
HUVECs + ZIKV	5.75 ± 0.46
PBMCs	9.35 ± 0.54
PBMCs + ZIKV	8.036 ± 0.67
PMNs	8.69 ± 0.54
PMNs + ZIKV	3.97 ± 0.71

HUVECs were cultured onto transwell plates and were mock-treated or infected with ZIKV. Afterwards, the medium was removed and human PBMCs or PMNs were added. Then, the FITC dextran transmigration across the membrane was calculated as the ratio of the upper to the lower chambers, in units/mL fluorescence.

**Table 2:** Quantification of pro-inflammatory cytokines.

Samples	ANALYTES (pg/mL)				
	GM-CSF	IL-1b	IL-6	INF-γ	TNF-α
HUVECs	-	<0.13 *	28.08	-	<1.14 *
HUVECs + ZIKV	-	<0.13 *	24.93	-	<1.14 *
PMNs	-	0.97	47.99	-	<1.14 *
PMNs + ZIKV	4.51	3.26	162.92	-	3.08
PBMCs	-	0.57	41.9	-	<1.14 *
PBMCs + ZIKV	-	<0.13 *	34.82	-	<1.14 *

Quantification of pro-inflammatory cytokine levels in the supernatants of the upper compartment of transwells, collected with 0.1 MOI 48 h p.i. * Value was extrapolated beyond the standard range.

## Data Availability

All data supporting reported results of this work are available from the corresponding author (Cusi, M.G., mariagrazia.cusi@unisi.it), upon reasonable request.
